# “Optochemistry”, when chemistry meets optics

**DOI:** 10.1080/14686996.2026.2665926

**Published:** 2026-05-12

**Authors:** Yohei Yamamoto, Soh Kushida

**Affiliations:** a Department of Materials Science, Institute of Pure and Applied Sciences, University of Tsukuba, Tsukuba, Japan; b Tsukuba Research Center for Energy Materials Science (TREMS) and Hydrogen Boride Research Center, Tsukuba Institute for Advanced Research, University of Tsukuba, Tsukuba, Japan

**Keywords:** Optochemistry, self-assembly, microstructure, optical resonator, microlaser, sensing, optical logic, light-matter strong coupling

## Abstract

In this review, we propose a term, ‘optochemistry’, a combination of optics and chemistry. In comparison with photochemistry that relates to photon aspect of light, optochemistry is a chemistry that relates to optics in which light is considered as wave or beam. Optochemistry and its applications include optical fibers, optical waveguides, organic resonators, lasers, organic polaritonics, light-matter strong coupling, optical vortices, optical manipulation, ultra-high sensitivity optical sensing, and so forth, that relate to the characteristics of molecules and polymers with chirality, spins (radicals), helices, supramolecules, semiconductors etc. The authors expect that the new-and-old optics and chemistry meet and generate a new field of research, and the term ‘optochemistry’ will gain acceptance to the society in the field of science and technologies.

## IMPACT STATEMENT

We propose the term “optochemistry”, where the wave character of light is combined with characteristic chemical materials, which will be beyond photochemistry where light is treated as photon, energy particle.

## IMPACT STATEMENT

**Figure:**
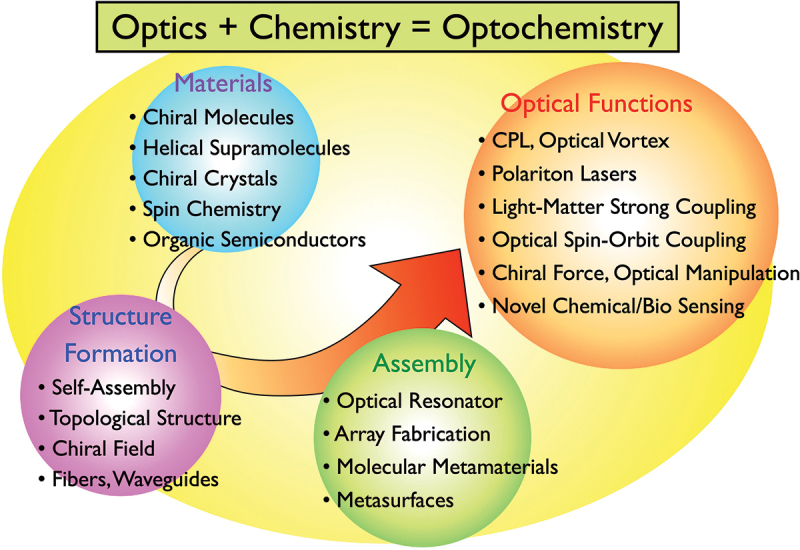

## 1. Optochemistry

In this review article, we propose the term ‘Optochemistry’, a portmanteau combining ‘optics’ and ‘chemistry’. The difference between photochemistry and optochemistry is described below: Photochemistry primarily treats light as photons (energy particles) and investigates phenomena such as light absorption, emission, excitation energy transfer, photoinduced electron transfer, and various photoreactions including photocatalysis and photoisomerization [1]. In contrast, optics treats light as rays or waves [2]. Although optics and photonics cannot be clearly distinguished, typical applications of optics include optical fibers, optical waveguides, optical resonators, lasers, and optical vortices. As optics is the study of light itself, it does not strictly require the presence of matter; however, recently, the emergence of a new field of optics directly reflects the diverse characteristics of materials.

Historically, the materials used in optics have been predominantly inorganic such as metals, ceramics, and silica. Although the application scope of organic and polymer materials remains limited, specific technologies such as polymer optical fibers composed of poly(methyl methacrylate) (PMMA) [3] and liquid crystalline (LC) molecules [4] are widely used. Nevertheless, organic materials possess unique structural and functional attributes – including chirality, radical, helicity, supramolecular assembly, and semiconducting properties – some of which are already being integrated into optical applications. Furthermore, concepts from topology, which has long been that debated in mathematics and physics is increasingly being introduced into the field.

**Figure 1.**
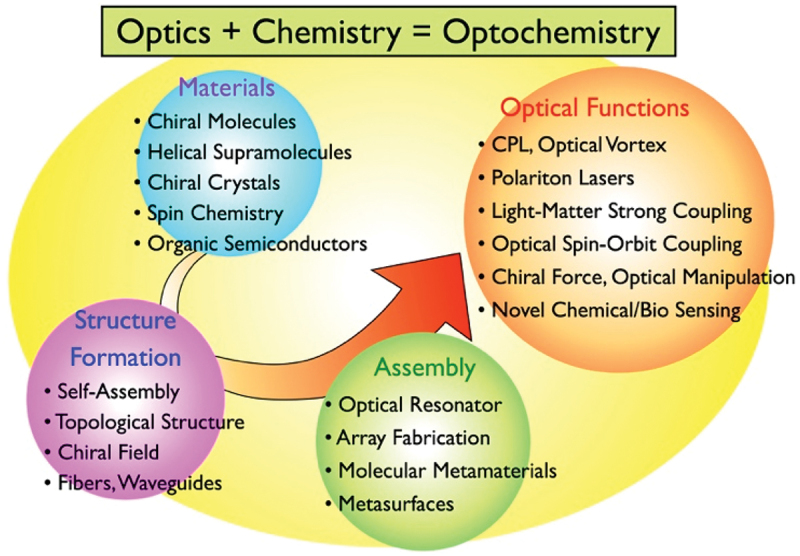
Conceptual diagram of Optochemistry.

Given the pioneering developments in areas such as light-matter strong coupling and optical vortices, we propose the collective term ‘Optochemistry’ to describe these intersecting domains (Figure 1). So far, the term ‘Optochemistry’ has been used in a few papers. Li *et al*. proposed optochemistry in their Perspective paper as chemistry using ultrashort pulse structured laser that selectively undergoes chemical reactions [5]. Also, the term optochemistry is used for application of photochemistry like photochromism and photocleavage to biochemistry and bio-optics field [6,7]. On the other hand, in this review, we use ‘optochemistry’ as much broader meaning: Chemistry that strongly relates to light with the aspect of wave or beam, and optics that utilize newly appeared chemistry. This can be viewed as one of the next evolutionary steps in photochemistry. Under the framework of optochemistry, we outline the precision synthesis of microstructures composed of organic and polymer materials and their subsequent applications in optics.

## 2. Self-assembly of π-conjugated organic molecules and polymers

**Figure 2.**
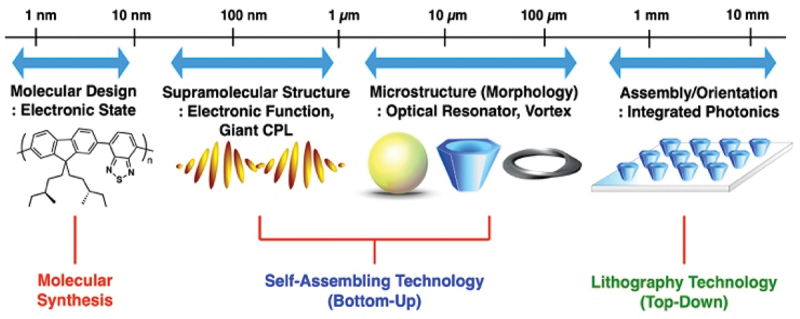
Schematic illustration of structural control and functions at each hierarchical level. Reproduced from ref 5. Copyright 2023 American chemical Society.

In the application of supramolecularly assembled materials, precise structural control across multiple hierarchical levels is of paramount importance (Figure 2) [8]. Specifically, the precise control of the aggregated structures is essential for the fabrication of micrometer-scale optical resonators from organic materials. The microfabrication of semiconductors has typically relied on ‘top-down’ lithographic processes. While this approach enables the formation of extremely fine structures at the nanometer scale, it necessitates multi-step fabrication procedures with highly expensive equipment. In contrast, chemists have explored ‘bottom-up’ approaches utilizing molecular self-assembly. The formation of precise molecular aggregates and integrated structures through self-assembly offers a streamlined, cost-effective, and energy-efficient alternative, leading many contemporary researchers to pursue material construction via the bottom-up methodologies.

**Figure 3.**
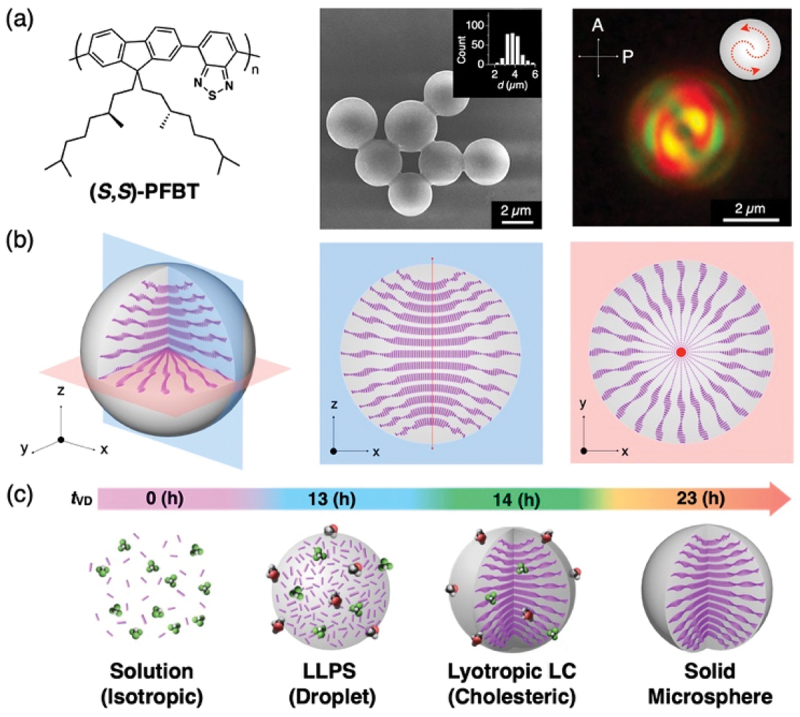
(a) Molecular structure of (S,S)-PFBT and electron and polarized optical microscopy images of the resulting microspheres. (b) Schematic illustration of the internal molecular alignment within the microsphere. (c) Schematic representation of the formation process of the twisted bipolar microsphere. Reproduced from ref 7. Copyright 2021 American chemical Society.

Our group began studying on the self-assembly of π-conjugated polymers around 2011. Through various investigations, we demonstrated that introducing bulky moieties into the side chains induces a twist in the polymer backbone. This twist inhibits π-stacking of the conjugated planes and suppresses crystallization, resulting in the formation of amorphous microspheres [9]. When a poor solvent is gradually introduced into a polymer solution via the vapor diffusion method, liquid-liquid phase separation occurs, leading to the formation of high-concentration polymer droplets (Figure 3(c)) [10]. As the proportion of the poor solvent increases further, the polymer aggregates to form solid spheres. By selecting appropriate polymers and precisely controlling the aggregation process, it is possible to fabricate not only simple spheres but also higher-order architectures, such as ‘colloidal molecules’ – complexes of interconnected spherical structures [11]. Furthermore, through the refined control of these self-assembling processes, the formation of non-spherical geometries (e.g. oblate spheroids, disks), topological structures (e.g. toroids), and spiral architectures has been reported [12,13].

Recently, we discovered unique microspheres with a topological internal structure known as a ‘twisted bipolar (TB)’ configuration, formed through the self-assembly of chiral conjugated polymers (Figure 3(a)) [10]. While such structures are often observed in LC droplets, this marked the first observation of such a configuration in a solid microsphere. Regarding the molecular orientation, the polymer backbones align unidirectionally at the central core, while forming a cholesteric helical arrangement toward the equatorial direction, characterized by structural singularities known as topological defects at the poles (Figure 3(b)). This architecture is analogous to the internal structure of a vertically bisected apple or pineapple. During the formation of this TB structure, a lyotropic liquid crystal phase emerges from the liquid-liquid phase separation, which eventually solidifies into a sphere as the solvent evaporates (Figure 3(c)). Although thin films of this polymer exhibit giant circularly polarized luminescence (CPL) with a dissymmetry factor (*g*-value) as high as 1.0 [14], we clarified that the microspheres exhibit angle-dependent CPL characteristics (*g* ~0.2–0.5) with the anisotropy ratio as high as 2.5, owing to the cholesteric orientation toward the equator.

Through the self-assembly of organic molecules, the formation of ‘skeletal crystals’ – microcrystals possessing concave surfaces – is identified [15]. Generally, when crystals grow under thermodynamic equilibrium, the resulting morphology is bounded by flat planes, forming convex polyhedra. Conversely, when crystallization occurs under solute diffusion-limited conditions, concave polyhedral crystals can be generated, as typically observed in snowflakes and bismuth crystals. However, since this is a kinetic growth process, precise control of the shape, size, and orientation of the crystals remains a significant challenge.

**Figure 4.**
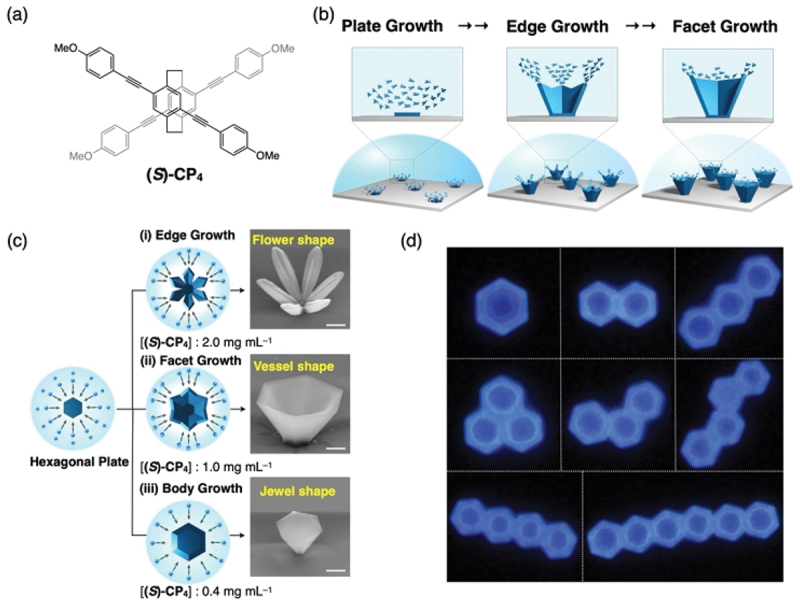
(a) Molecular structure of planar chiral cyclophane (S)-CP4. (b) Schematic illustration of the formation process of bowl-shaped microcrystals. (c) SEM images of microstructures formed at different initial concentrations. (d) Fluorescence microscopy image of polycyclic aromatic hydrocarbon-like structures formed by the concatenation of bowl-shaped microcrystals. Reproduced from ref 12. Copyright 2022 AAAS.

During our investigation into the self-assembly of planar chiral cyclophane molecules, we discovered the formation of single crystals with a hexagonal pyramidal shape and a concave center. Furthermore, these crystals exhibited remarkably low size dispersity. The fabrication process is straightforward: a solution of (*S*)-CP_4_ dissolved in hot ethanol is cast onto a quartz substrate (Figure 4(a)). Due to the low solubility of the molecules in ethanol, the evaporation of the solvent and the subsequent temperature drop immediately trigger the simultaneous formation of hexagonal plate-like crystal nuclei on the substrate surface. Because the solution concentration drops rapidly thereafter, no further nucleation occurs, and the crystals grow at a uniform rate from the existing nuclei. Consequently, solute diffusion-limited growth leads to preferential development at the crystal edges (Figure 4(b)). High initial concentrations yield flower-shaped crystals where only the edges grow, while lower concentrations result in filled-in structures (Figure 4(c)). Given the uniformity of the crystal sizes, the crystals can also interconnect to form micro-architectures reminiscent of polycyclic aromatic hydrocarbons (Figure 4(d)).

## 3. Microspherical organic optical resonators and lasers

In contemporary materials science, organic light-emitting diodes (OLEDs) have reached successful commercialization with OLED-equipped televisions and smartphones now ubiquitous in the global market. Furthermore, the development of next-generation OLED materials remains a highly active field of research. One of the primary frontiers for the next generation of organic photonics devices is the realization of organic semiconductor laser diodes (OSLDs). In Japan, research and development of current-driven organic lasers have been spearheaded by groups such as Adachi and colleagues at Kyushu University [16]. However, a significant challenge remains: the high current densities required to reach the lasing threshold often lead to the degradation of the organic materials before lasing can be generated. Overcoming this stability issue is thus of paramount importance. Conversely, optically pumped organic lasers were realized as early as the 1990s. Following the seminal observations of laser oscillation in π-conjugated polymers via optical pumping by Friend, Heeger, and others [17,18], numerous studies have been reported, the early progress of which is detailed in a comprehensive review by Samuel et al. [19]. More recently, indirect current-driven organic lasing induced by OLEDs has also been demonstrated [20].

**Figure 5.**
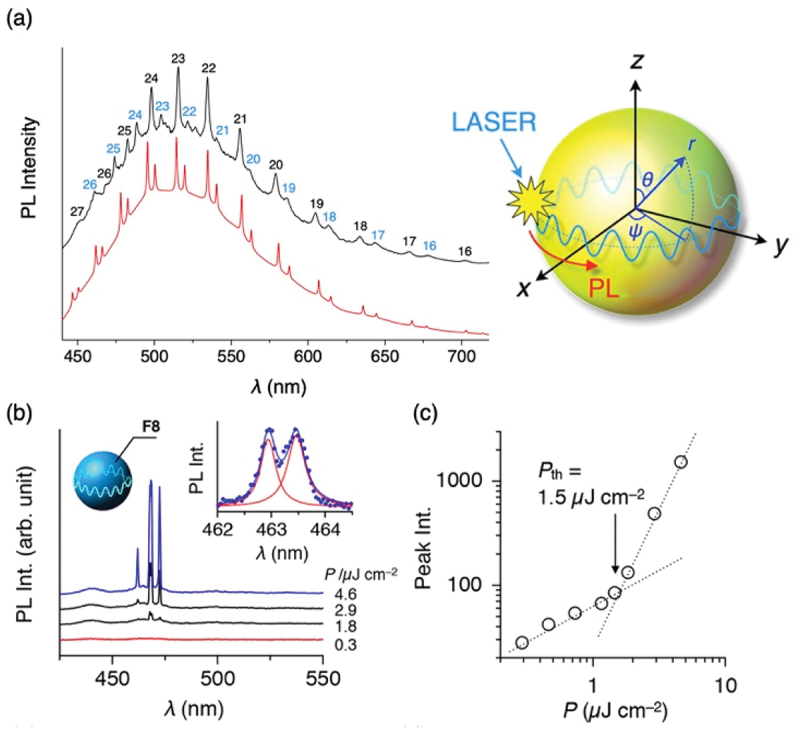
(a) WGM emission from a microsphere optical resonator. (b) Laser oscillation from a polyfluorene microsphere. Panel a reproduced from ref 18. Copyright 2014 Springer Nature. Panel b reproduced from ref 19. Copyright 2017 Wiley.

As for the organic microlasers, Zhao et al., summarized optically pumped organic dye-doped microlasers and their array formation for optoelectronic applications [21]. The authors in the present review focus on microresonators and lasers from a single π-conjugated polymer microsphere fabricated via the methods described in the previous section. Our results revealed whispering gallery mode (WGM) emission, in which the luminescence is confined and resonates within the spherical cavity (Figure 5(a)) [22]. In this phenomenon, light generated within the surface layer of the sphere (typically within 100 nm-thick) undergoes total internal reflection at the polymer-air interface. As the light circulates, it undergoes self-interference, causing constructive interference at specific wavelengths and resulting in the appearance of periodic resonant peaks in the emission spectrum. Under intense excitation using a femtosecond (fs) laser, a population inversion is established, leading to light amplification via stimulated emission and subsequent laser oscillation (Figure 5(b)) [23]. Furthermore, by utilizing luminescent photoisomerizable molecules as the gain medium, the dynamic switching of WGM resonances was demonstrated [24]. By forming hemispherical arrays through self-assembly on substrate surfaces, the WGM ‘fingerprints’ work as physical unclonable functions (PUFs).

## 4. Organic microcrystalline lasers

**Figure 6.**
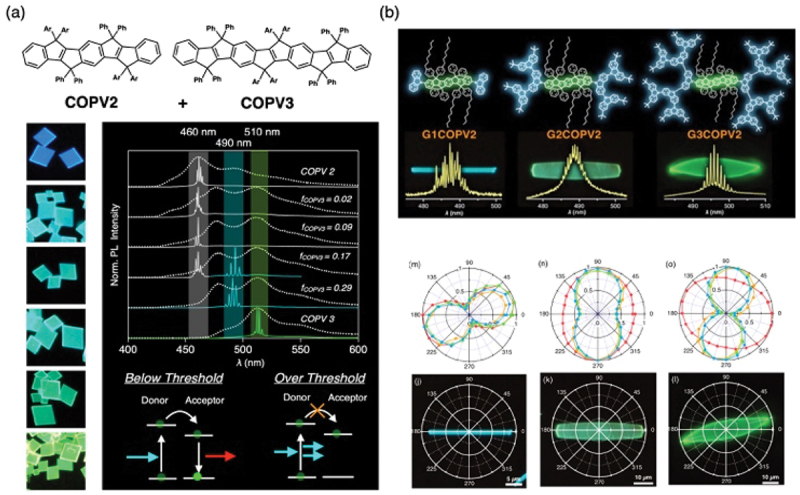
(a) WGM laser oscillation from a COPV microcrystal. (b) Angle-anisotropic emission and laser oscillation spectra from dendron-modified COPV. Panel a reproduced from ref 22. Copyright 2018 American chemical Society. Panel b reproduced from ref 24. Copyright 2020 Wiley.

Optical resonance occurs within microcrystals, where light is confined and undergoes self-interference. Carbon-bridged oligo (phenylenevinylene) (COPV), developed by Tsuji and colleagues, is a laser dye characterized by high photostability due to its rigid π-conjugated plane (Figure 6(a)) [25]. When a solution of COPV is cast onto a substrate under appropriate conditions, square plate-like crystals are deposited. Laser oscillation is observed upon fs-laser excitation of these crystals [26]. In a single crystal formed by adding COPV3 (energy acceptor) into a host crystal of COPV2 (energy donor), it was initially expected that COPV2 would harvest light and transfer energy to COPV3, leading to a population inversion and subsequent lasing from COPV3. However, the rate of laser oscillation from COPV2 was found to be approximately 20 times faster than the energy transfer rate; consequently, lasing from COPV2 before energy transfer could take place. In contrast, recent work using a COPV2-COPV3-COPV2 linked molecule has successfully achieved laser oscillation from COPV3 via efficient and ultrafast intramolecular energy transfer [27]. Furthermore, macromolecules consisting of a COPV core flanked by carbazole dendrons at both termini also self-assemble into microcrystals (Figure 6(b)) [28]. In this system, the dendrons act as light-harvesting antenna, transferring energy to the COPV core that induces lasing.

The use of flexible microcrystals as optical resonators allows for the detection of structural deformations through shifts in resonance peaks. Cyano-substituted oligo(phenylenevinylene), developed by Hayashi et al., exhibits weak interactions between stacked molecular columns, resulting in the formation of rod-shaped crystals that can bend flexibly [29]. Since this structural deformation modulates the Fabry-Perot (F-P) mode resonance caused by reflection at the crystal facets, it enables the sensing of mechanical deformation [30]. Further organic crystalline microlasers are summarized by Wang et al. in their account paper [31].

## 5. High-sensitivity sensing using organic microresonators

Sensors using optical resonators are characterized by their resonant mode shift induced by the changes in structure or refractive index, offering extreme sensitivity. Sensing with inorganic optical resonators typically detects changes in the refractive index accompanying the adsorption/desorption of molecules or gases onto the surface of the resonator. In contrast, organic and polymeric materials are structurally flexible; the penetration of gases or molecules into the interior of the resonator induces more significant modulation of the resonant modes, thereby enabling ultra-high sensitivity sensing [32]. Furthermore, unlike electrical sensing, optical resonator-based sensing does not require electrodes, allowing for non-contact and remote monitoring.

**Figure 7.**
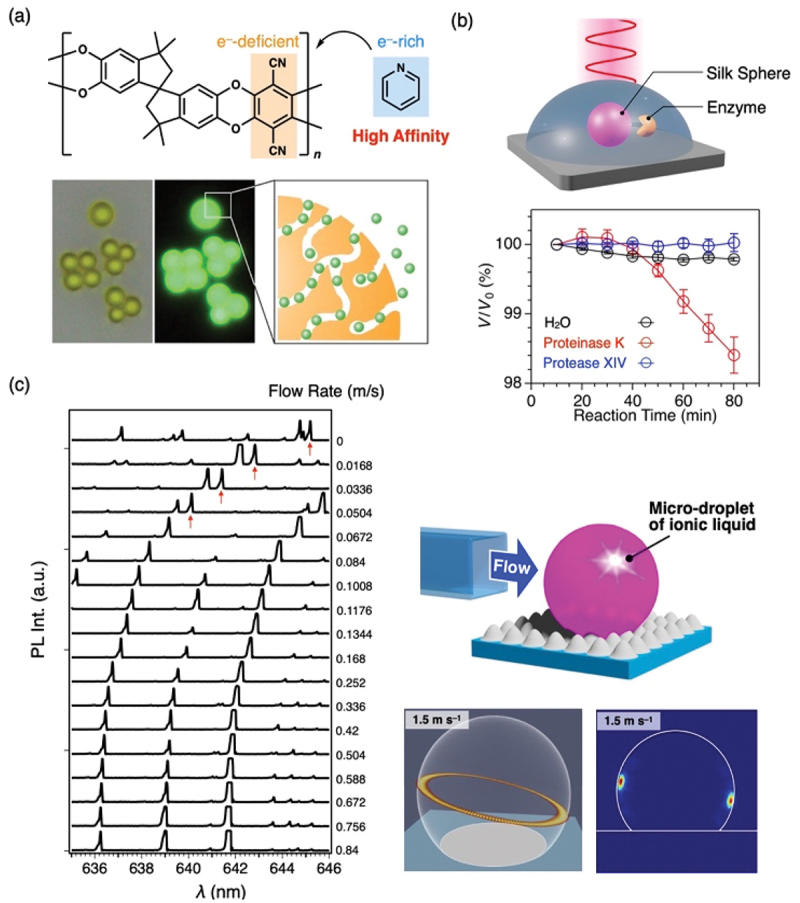
(a) Schematic illustration of voc sensing using a PIM-1 microsphere. (b) Monitoring of enzymatic silk degradation. (c) Breeze sensing using an ionic liquid droplet. Panel a reproduced from ref 28. Copyright 2022 American chemical Society. Panel b reproduced from ref 30. Copyright 2023 royal Society of chemistry. Panel c reproduced from ref 31. Copyright 2023 Wiley.

Microspheres formed from a luminescent polymer with intrinsic microporosity (PIM-1) readily adsorb volatile organic compounds (VOCs) within their pores (Figure 7(a)). The resulting increase in refractive index leads to a redshift of the resonance peaks [33]. For pyridine vapor, which shows the highest sensitivity, the detection limit is as low as 470 ppb, with a sensitivity reaching 0.40 nm ppm^−1^.

Microspheres composed of biopolymers doped with fluorescent dyes also function as optical resonators. For instance, microspheres fabricated from highly hygroscopic proteins such as silk fibroin or hyaluronic acid undergo significant changes in size and shape in response to humidity fluctuations. These changes cause pronounced shifts in resonance peaks, allowing the spheres to function as humidity sensors [34]. Additionally, by utilizing the enzymatic degradation of proteins, high-sensitivity monitoring of the initial stages of proteolysis has been demonstrated (Figure 7(b)) [35].

Anemometric (wind) sensing is also possible using droplet lasers composed of non-volatile ionic liquids (Figure 7(c)) [36]. Laser oscillation is observed by fs-excitation of a droplet formed by dispersing fluorescent dyes in an ionic liquid and depositing them onto a superhydrophobic surface via casting or inkjet printing method. Droplets are far more susceptible to structural deformation than solids; even a gentle breeze of ~0.01 m/s can deform the structure, resulting in a detectable shift in the laser peaks. Furthermore, by applying an external electric field to the droplet, the subtle deformation of its shape increases the lasing threshold, enabling the switching of laser oscillation via an ON/OFF external electric field [37].

## 6. Crystalline organic optical waveguides and optical logic gates

**Figure 8.**
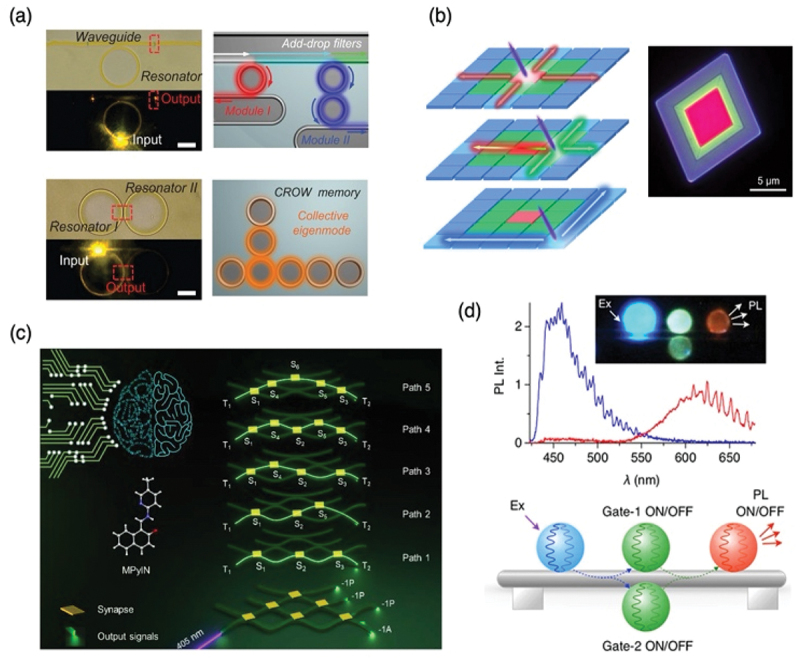
(a) Optical logic gates via concatenated microring resonators. (b) Optical gates using multi-color emitting organic microcrystals. (c) Neural network optical gates based on flexible organic crystal fibers. (d) Logic gates utilizing photo-switchable microspheres. Panel a reproduced from ref 33. Copyright 2015 AAAS. Panel b reproduced from ref 34. Copyright 2025 AAAS. Panel c reproduced from ref 35. Copyright 2025 Wiley. Panel d reproduced from ref 36. Copyright 2021Wiley.

Research is also progressing on the construction of optical waveguides and logic gates using nano- to micrometer-scale polymer or organic crystal fibers. Zhao *et al*. constructed a photonic integrated circuit (PIC) by integrating organic ring resonators using an inkjet printing method (Figure 8(a)) [38]. Their construction strategy allowed to design a complex assembly of one-dimensional waveguide and resonator components for light signal filtering and optical storage toward the large-scale on-chip integration of microscopic photonic units. They developed a scheme for soft photonic integration that motivates further studies on organic photonic materials and devices.

Wang *et al*. realized multi-mode logic gates for in-plane optical waveguiding within plate-like crystals (Figure 8(b)) [39]. Their cascaded strategy demonstrates the hierarchical assembly of organic lateral heterostructure (OLH) with in-plane multicolor emission, from red-blue and red-green to lateral red-green-blue (RGB). The tunable regions of 2D OLHs are realized by synergistic effects of the molecular doping method and the photo-induced oxidation route during the epitaxial growth process. The obtained OLHs can actively achieve full-spectrum light transport from 420 to 720 nanometers depending on different excitation positions and thus function as multimode RGB signal converters.

Chandrasekar *et al*. utilized fibers composed of flexible organic crystals to construct optical gates that mimic neural networks (Figure 8(c)) [40]. They present an interconnected, four-layered organic crystal optical waveguide architecture that mimics an artificial neural networks. This structure is constructed from pseudo-plastic organic crystals using an atomic force microscopy cantilever tip-based micromanipulation technique. By strategically selecting four crystal waveguides of varying lengths, bending them into serpentine-like forms, and integrating them hierarchically, they create interconnected, neuron-like optical waveguides with six optical synapses, which enable parallel transmission of passive optical signals through evanescent coupling across multiple paths within the waveguides. The feedforward mechanism allows the synapses to split the input optical signal into four diverging signals with different magnitudes.

Our group also demonstrated the construction of optical logic gates by positioning light-switchable optical resonators on an optical microfiber (Figure 8(d)). By utilizing three kinds of resonators with different luminescent colors (blue, green and red) and green one is photoswitchable, light energy transfer cascade is switched by turning on and off the green fluorescence microresonator [41]. By combining these optical resonators on a polystyrene fiber, AND/OR optical logic gates are demonstrated. Further, spider silk fibers are utilized as the optical fiber, NOT gate and much more complicate gate operations are performed [42].

## 7. Light–matter strong coupling in organic resonators

The discussions thus far have focused on the optical properties of systems where light and matter exist independently. In recent years, the phenomenon of ‘light-matter strong coupling’, in which light and matter are hybridized, has attracted significant attention in both fields of physics and chemistry. The early review was summarized by Ebbesen, where the hybrid light-matter states in a molecular and material science perspective are described [43].

**Figure 9.**
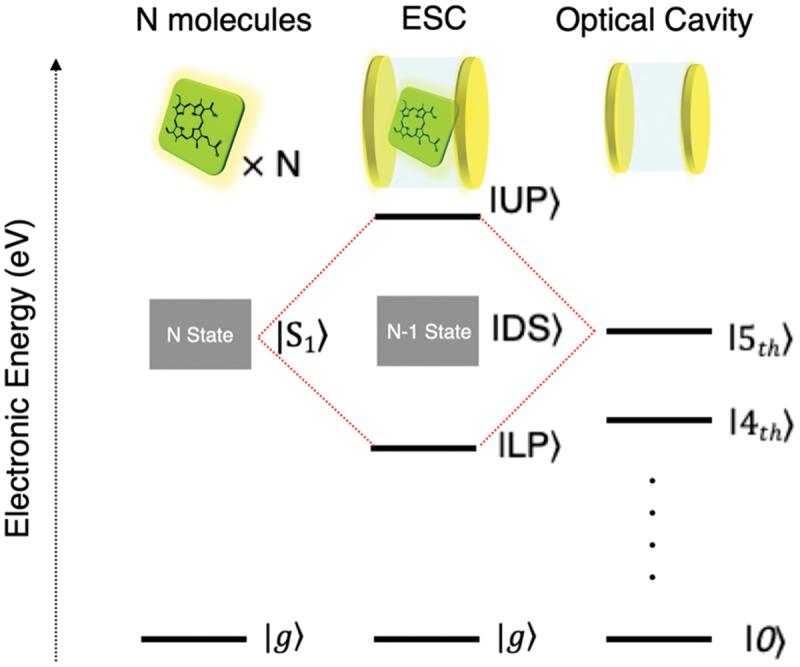
Schematic illustration of light – matter strong coupling. Reproduced from ref 40. Copyright 2025 American chemical Society.

Strong coupling refers to a state in which the transition dipole moment of a molecule and the optical mode within a resonator interact so intensely that the rate of energy exchange between them exceeds the dissipation rates of both photons and molecules. Under these conditions, the energy states undergo Rabi splitting into two new hybrid states: the upper polariton (UP) and the lower polariton (LP), as illustrated in Figure 9. Between the UP and LP states, there exist dark states (DS), which are optically forbidden states, where *N* represents the number of molecules involved in the coupling. The resulting quasi-particles, termed polaritons, exhibit the characteristics of both excitons and photons, behaving as a novel hybrid state. Various unique phenomena have been observed in such hybrid light-matter systems.

In the context of chemistry, the seminal work by Ebbesen *et al*. demonstrating the modulation of reaction kinetics in photoisomerization molecules under strong coupling attracted widespread interest and gave rise to the field of polaritonics which focuses on molecular behavior in hybrid light-matter states [44]. Recent reports have even suggested that material properties can be modulated without external light excitation [45]. However, the underlying mechanisms remain under debate and have yet to be unified.

While the possibility of ‘property control without photoexcitation’ is one of the primary attractions of polaritonics, we emphasize that focusing on photoexcitation and the subsequent energy relaxation dynamics will also be essential for gaining fundamental insights into polaritonics. In particular, although the modulation of polariton decay kinetics via strong coupling has been reported by several research groups, their interpretations remain inconsistent at several points.

To date, most analyses of exciton-polariton photodynamics have relied on fluorescence lifetime measurements and transient absorption spectroscopy (TAS), primarily targeting polymer-dispersed films or solid-state materials. In contrast, achieving exciton strong coupling (ESC) in a liquid state and measuring its dynamics remains an underexplored frontier due to technical difficulties. We realized ESC in a liquid phase using high-concentration solutions of a chlorophyll-based dye, Ce06, and characterized its post-excitation dynamics [46]. This was achieved by confining the solution in a custom-built microfluidic Fabry – Perot (FP) resonator, with the cavity thickness precisely adjusted to 1.07 µm. Strong coupling between the Q-band of Ce06 and the 5th-order cavity mode resulted in a distinct Rabi splitting of 0.13 eV, indicating a sufficiently high coupling strength between the molecules and the cavity mode.

TAS using ultrashort pulse lasers revealed that the time constant under ESC is totally distinct from those observed under non-resonant conditions. In a control system without a resonator at the same concentration, components of approximately 150 ps and 1.58 ns, that are attributed to excimer formation and an excimer lifetime, respectively, were observed. Under ESC, however, a fast component of approximately 50 ps emerged alongside the 150 ps component. We interpret this fast 50 ps component as a relaxation from the LP to the DS. According to the ‘entropy-reordered’ free energy landscape theory proposed by Scholes *et al*., numerous dark states are formed in the ESC state, which become lower in free energy than the LP due to entropic gains [47]. Since our system utilizes a high-order cavity mode (the 5th mode) and involves a large number of molecules (*N*), the density of states (DoS) for the DS increases, leading to a strong free energy inversion driven by entropic effects. Under such conditions, rapid LP-to-DS relaxation is a predicted natural process. Supporting this, no luminescence was observed from the LP despite its hybrid photon-molecule nature, suggesting the existence of a dominant non-radiative decay pathway – specifically, the transition to the optically forbidden DS. However, definitive proof requires further exploration of mode-dependent photodynamics, which is currently ongoing.

Furthermore, to capture the anisotropic relaxation characteristic of liquids, we utilized a self-developed Resonant Optical Kerr Effect (ROKE) spectroscopy method. We demonstrated that ESC significantly accelerates the relaxation of photoinduced anisotropy (molecular orientation) [48]. Specifically, anisotropy is lost in only 15.5 ps when the LP state is excited, and in only 9.9 ps upon UP excitation. These rates are far faster than the molecular rotational relaxation time (170 ps) or the energy transfer/excimer formation times (150–200 ps). This result suggests that ESC opens new energy transfer pathways, leading to a rapid transition to collective DS. These findings clarify that the interaction between photons and molecules in the ESC state in a liquid exhibits dynamics beyond conventional understanding, providing new design principles for controlling photochemical reactions and energy transfer processes in the liquid phase. We believe these developments – particularly the influence of the relationship between the cavity mode number and the molecular count on the free energy landscape – will be of great significance for the future design of functional optical materials.

## 8. Conclusion and future perspectives

In this review article, we have proposed ‘Optochemistry’ as a nascent interdisciplinary domain that integrates the principles of chemistry and optics. Photonics has flourished since the 20th century, treating light as energy particles called photon. In contrast, optics possesses a much longer history, tracing its origins back to Newton and even earlier, as a fundamental inquiry into the nature of light. At first glance, it may seem paradoxical that ‘Optochemistry’ is now emerging, following the long-standing development of ‘Photochemistry’, which combined light and chemistry. However, considering the recent significant progress in understanding the interactions between matter and light as a wave, this historical sequence appears entirely logical.

Although not discussed in detail within this review, the integration of concepts such as topology, optical vortices, and other multifaceted intersections with physics and biology promises to evolve this field into an even more compelling area of research. We eagerly hope that this contribution serves as a catalyst for ‘Optochemistry’ – a domain that has long been latent but never formally defined – to permeate academic and societal spheres, eventually establishing itself at the forefront of modern science.
